# The Plate, the Policy, and the Price: Reframing Nutrition Management in Healthcare

**DOI:** 10.3390/nu18142265

**Published:** 2026-07-10

**Authors:** Alvin Wong

**Affiliations:** Department of Dietetics, Changi General Hospital Singapore, Singapore 529889, Singapore; alvin_wong@cgh.com.sg

## 1. Introduction

Nutrition management in healthcare is frequently described in clinical terms: identifying nutritional risk; estimating requirements; prescribing diet, food for special medical purposes, or nutrition support; and monitoring response [1]. While technically correct, this description is incomplete. In practice, nutrition management is a complex, system-dependent intervention shaped not only by clinical need, but also by patient behaviour, caregiver capacity, food access, organisational workflow, workforce availability, financing arrangements, and policy infrastructure [2,3].

This Special Issue, “The Economics, Environment and Policies That Influence Nutrition Management in Healthcare”, examines these interacting determinants and is grounded in the premise that effective nutrition care cannot be reduced to the provision of biochemically adequate nutrients alone. Rather, it comprises a multicomponent process of assessment, prioritisation, decision-making, delivery, adherence support, monitoring, and adaptation across clinical, community, and household settings [3,4,5,6]. This broader framing is increasingly necessary in the context of population ageing, multimorbidity, widening social inequality, constrained workforce capacity, rising healthcare expenditure, and a continued shift of care from hospitals into community and home settings. Accordingly, the relevant question is no longer whether nutrition interventions work, but for whom, under what circumstances, at what cost, through which mechanisms, and with what consequences for patients, caregivers, health services, and society [4,7].

The contributions in this Special Issue collectively show that nutrition care succeeds or fails at the interface between nutrient requirements and real-world conditions. Across themes including economic modelling, energy requirement estimation, food insecurity, home enteral nutrition, behavioural determinants of healthy eating, and measurement of nutrition- and exercise-related knowledge, attitudes, and practices, they make a consistent case: nutrition interventions cannot be fully understood without examining the systems in which they are delivered.

## 2. Economic Value, Resource Allocation, and the Cost of Implementation

Economic considerations are central to nutrition management because healthcare systems must prioritise interventions that are effective, feasible, affordable, and scalable. However, economic evaluation should extend beyond projected cost savings to consider cost-effectiveness under modelled assumptions, budget impact in real-world settings, patient and caregiver affordability, service opportunity costs, and the distribution of benefits and burdens across population groups [8].

Wakayama and colleagues used a Markov cohort simulation model to estimate the projected health and economic impact of increasing milk consumption to meet recommended dairy intake in Japan [Contribution 1, Wakayama et al., 2026]. Their model suggested that increasing milk consumption to recommended levels could reduce stroke incidence, stroke-related mortality, and national healthcare expenditure. This study illustrates how dietary recommendations can be evaluated as population-level health policies rather than solely as individual-level lifestyle advice.

This study [Contribution 1, Wakayama et al., 2026] also demonstrates an important boundary of policy modelling. Projected healthcare savings do not, by themselves, constitute an implementation strategy. Sustaining population dietary change requires coordinated investment in education, subsidies, food systems, procurement, product availability, culturally appropriate messaging, and equity-focused food access [9].

The economic logic of nutrition care also applies at the bedside. Kua and colleagues developed novel resting energy expenditure prediction equations for multi-ethnic Asian older adults with multimorbidity [Contribution 2, Kua et al., 2025]. Their study addressed a practical clinical problem, where standard predictive equations may perform poorly in populations that differ in age, ethnicity, multimorbidity, or body composition compared to their development cohorts. The subsequent recalibration study [Contribution 3, Kua et al., 2026] further demonstrated that standard predictive equations may systematically misestimate requirements in specific body mass index categories, particularly amongst underweight and obese older adults.

These studies are important because precision in nutrition management does not necessarily require expensive technology for every patient. Population-specific, validated, clinically usable tools may improve nutritional care planning while remaining feasible for routine service delivery. Inaccurate energy estimation may increase the risk of underfeeding or overfeeding and may compromise the application of a dietetic intervention, which may implicate recovery, length of stay, readmission, and resource utilisation.

## 3. Environments, Food Access, and the Limits of Clinical Prescription

Nutrition management also depends on environments that shape what patients can obtain, prepare, tolerate, afford, and sustain. These include hospitals, homes, community services, food assistance systems, supply chains, regulation, and family care arrangements [4]. Even clinically appropriate prescriptions may fail when the care environment does not support implementation [10].

Breik and colleagues’ narrative review of home enteral nutrition in Australia, with a focus on blended tube feeding, illustrates this environmental complexity [Contribution 4, Breik et al., 2025]. Home enteral nutrition is not simply a route of feeding; it is a care ecosystem involving funding models, clinical guidelines, equipment supply, professional confidence, caregiver capability, patient preference, food safety, and advocacy [11]. The renewed interest in blended tube feeding adds further complexity because it intersects with autonomy, cultural identity, family routines, safety concerns, and inequities in access to professional support [12].

Lim and colleagues’ study of food accessibility and nutritional outcomes among food-insecure pregnant women in Singapore provides another example of the limitations of purely clinical assumptions [Contribution 5, Lim et al., 2025]. Participants were, on average, overweight before pregnancy, yet had suboptimal dietary intake and biochemical evidence of nutritional inadequacy, including low vitamin D status. The qualitative findings showed how financial constraints, pregnancy symptoms, food supply management, and the perceived inadequacy or misalignment of food assistance shaped actual dietary behaviour. This challenges the assumption that national food availability necessarily translates into household nutritional adequacy. It also illustrates why nutritional risk screening should not rely on anthropometry alone. In food-insecure populations, excess body weight and micronutrient inadequacy can coexist. Policy responses must therefore address diet quality, cultural suitability, affordability, food literacy, and the practical acceptability of food assistance rather than food quantity alone [4,7,13].

## 4. Behavioural Mechanisms and the Conditions for Sustained Dietary Change

Nutrition interventions often assume that information leads to behaviour change. However, knowledge alone is rarely enough when patients face barriers related to cost, autonomy, symptoms, motivation, social norms, culture, food availability, and confidence to change [14]. Ma and colleagues extended the Theory of Planned Behaviour by incorporating intrinsic motivation as a mediator of healthy eating intentions among Chinese adults [Contribution 6, Ma et al., 2025]. Their findings indicate that attitudes and subjective norms influence motivation, while motivation and perceived behavioural control shape intention. This has direct implications for nutrition policy and intervention design—an example being that nutrition education alone is unlikely to be sufficient where autonomy, resources, social support, or perceived behavioural control are constrained [10].

Behavioural mechanisms are particularly important because many nutrition interventions depend on repeated actions outside direct clinical supervision [5,6]. These include food purchasing and preparation, adherence to supplement use and therapeutic diets/tube feeding routines, attendance at follow-up, and symptom management. Effective intervention design must address both internal determinants, including motivation and perceived control, and external conditions, including affordability, accessibility, social support, service availability, and culturally appropriate resources [3,5,6,14].

## 5. Measurement and the Evaluation of Complex Nutrition Interventions

Nutrition management targets clinical outcomes as well as knowledge, attitudes, motivation, adherence, caregiver capability, dietary behaviour, treatment burden, food security, and quality of life. Complex interventions cannot be evaluated appropriately when their mechanisms are poorly measured [2].

Hoe and colleagues’ scoping review of questionnaires assessing nutrition- and exercise-related knowledge, attitudes, and practices in cancer survivorship identified substantial heterogeneity in available measurement tools [Contribution 7, Hoe et al., 2025]. The review found 200 unique questionnaires, including 45 untitled study-specific instruments, many with limited psychometric evaluation. This lack of validated, standardised tools limits comparison, evidence synthesis, and understanding of intervention mechanisms, especially in cancer survivorship, where behaviours are shaped by fatigue, treatment effects, misinformation, fear of recurrence, cultural beliefs, access to guidance, and varied recovery trajectories [Contribution 7, Hoe et al., 2025].

Measurement limitations are not restricted to behavioural questionnaires. Several contributions in this Special Issue highlight the need for population-specific tools, better external validation, and more granular data. Predictive equations developed in one population may not generalise to another [Contribution 3, Kua et al., 2025]. Food assistance programmes designed around generic food provision may not address micronutrient adequacy or cultural acceptability [Contribution 5, Lim et al., 2025]. Behavioural models may perform differently across socioeconomic, ethnic, and health status groups [Contribution 6, Ma et al., 2025]. External validation across populations, care settings, and policy environments should therefore become standard practice in nutrition management research [Contribution 1, Wakayama et al., 2025; Contribution 4, Breik et al., 2025; Contribution 7, Hoe et al., 2025].

## 6. Policy as an Enabler or Constraint of Nutrition Care

Policy determines whether nutrition interventions are accessible, reimbursed, staffed, prioritised, measured, and sustained. Without policy alignment, evidence-based nutrition care may remain effective in principle but unavailable in practice [9]. The contributions in this Special Issue show that policy is not an external background factor: *it is part of the intervention system*.

In home enteral nutrition, funding models and clinical guidelines shape access to equipment, formulae, blended tube feeding support, and professional oversight [Contribution 4, Breik et al., 2025]. In food-insecure pregnancy, welfare policies and food assistance structures influence whether nutritional advice can be translated into adequate dietary intake [Contribution 5, Lim et al., 2025]. In population dietary modelling, dietary guidelines only become meaningful when coupled with feasible implementation mechanisms [Contribution 1, Wakayama et al., 2026]. In clinical energy estimation, the service-level adoption of validated tools determines whether evidence improves routine practice [Contribution 2, Kua et al., 2025 and Contribution 3, Kua et al., 2026].

## 7. Priorities for the Future of Nutrition Research in Healthcare

The contributions in this Special Issue reveal several priorities for the next phase of research. First, economic evaluation must move beyond projected healthcare savings. Future studies should measure implementation costs, workforce time, caregiver burden, patient out-of-pocket expenditure, resource utilisation and costs, long-term care use, and cross-sector consequences.

Second, nutrition intervention studies require stronger process evaluation. Many interventions combine assessment, counselling, supplementation, food service modification, caregiver training, behavioural support, and follow-up [3,4]. These components are often evaluated as bundled programmes, making it difficult to identify which components drive outcomes and which are redundant, infeasible, or poorly implemented. Future trials and service evaluations should measure reach, adoption, fidelity, dose delivered and received, acceptability, adaptation, and sustainability [15].

Third, measurement tools require stronger validation. Population-specific prediction equations, behavioural questionnaires, food security measures, patient-reported experience measures, and nutrition knowledge instruments should undergo external validation across ethnic groups, socioeconomic strata, disease states, and care settings.

Fourth, equity should be treated as a core analytical domain. Nutrition management is shaped by income, food access, education, language, culture, caregiving capacity, disability, health literacy, and eligibility for support schemes [4,7]. Research should examine not only effects, but also who benefits, who is excluded, who bears the cost, and whether policy mechanisms reduce or widen inequities.

Finally, nutrition research should integrate implementation science, health economics, behavioural science, and systems thinking [15]. These disciplines should not be added after efficacy has been established; they should inform intervention design from the outset. Nutritional care pathways should be co-designed with patients, caregivers, clinicians, service providers, and policy stakeholders. This is particularly important when interventions require sustained behaviour change, interprofessional coordination, or delivery across various settings [15].

## 8. Conclusions

The central message of this Special Issue is that nutrition management should be evaluated as a complex intervention embedded within complex systems. Improving nutrient prescription alone is insufficient. Better nutrition care depends on valid assessment tools, behaviourally informed interventions, trained professionals, adequate financing, supportive environments, culturally appropriate resources, and policies that enable sustained implementation. Nutrition must be part of healthcare management, not an afterthought. It involves a three-level approach of aligning nutrition guidance, policy, environmental, economic, and social determinants to improve health outcomes. Ultimately, nutrition management should be judged by whether systems enable the right intervention to reach the right person, in the right form, at the right time, and at a cost that patients, caregivers, and healthcare systems can sustain.

## References

[1] Cederholm T., Barazzoni R., Austin P., Ballmer P., Biolo G., Bischoff S.C., Compher C., Correia I., Higashiguchi T., Holst M. (2017). ESPEN guidelines on definitions and terminology of clinical nutrition. Clin. Nutr..

[2] Skivington K., Matthews L., Simpson S.A., Craig P., Baird J., Blazeby J.M., Boyd K.A., Craig N., French D.P., McIntosh E. (2021). A new framework for developing and evaluating complex interventions: Update of Medical Research Council guidance. BMJ.

[3] Wong A., Huang Y., Banks M.D., Sowa P.M., Bauer J.D. (2024). A Conceptual Study on Characterizing the Complexity of Nutritional Interventions for Malnourished Older Adults in Hospital Settings: An Umbrella Review Approach. Healthcare.

[4] Hickson M., Papoutsakis C., Madden A.M., Smith M.A., Whelan K. (2024). Nature of the evidence base and approaches to guide nutrition interventions for individuals: A position paper from the Academy of Nutrition Sciences. Br. J. Nutr..

[5] Riddle E., Munoz N., Clark K., Collins N., Coltman A., Nasrallah L., Nishioka S., Scollard T., Simon J.R., Moloney L. (2024). Prevention and Treatment of Malnutrition in Older Adults Living in Long-Term Care or the Community: An Evidence-Based Nutrition Practice Guideline. J. Acad. Nutr. Diet..

[6] Wong A., Bauer J.D. (2024). Addressing the malnutrition gap requires high-quality research, recognition of intervention complexity, and equitable implementation strategies. Hepatobiliary Surg. Nutr..

[7] Downer S., Berkowitz S.A., Harlan T.S., Olstad D.L., Mozaffarian D. (2020). Food is medicine: Actions to integrate food and nutrition into healthcare. BMJ.

[8] Fattore G., Federici C., Drummond M., Mazzocchi M., Detzel P., Hutton Z.V., Shankar B. (2021). Economic evaluation of nutrition interventions: Does one size fit all?. Health Policy.

[9] Mozaffarian D., Angell S.Y., Lang T., Rivera J.A. (2018). Role of government policy in nutrition-barriers to and opportunities for healthier eating. BMJ.

[10] Agurs-Collins T., Alvidrez J., ElShourbagy Ferreira S., Evans M., Gibbs K., Kowtha B., Pratt C., Reedy J., Shams-White M., Brown A.G. (2024). Perspective: Nutrition Health Disparities Framework: A Model to Advance Health Equity. Adv. Nutr..

[11] Ojo O. (2015). The challenges of home enteral tube feeding: A global perspective. Nutrients.

[12] Reilly C., Ross N., Watene S., Lindeback R., Coelho T., Krishnan U., Perez W.P., Chandrasekar N., Yap J., Breik L. (2025). A study of professional practices, attitudes and barriers to blended tube feeding in Australia and New Zealand. Nutr. Diet..

[13] Silva P., Araújo R., Lopes F., Ray S. (2023). Nutrition and Food Literacy: Framing the Challenges to Health Communication. Nutrients.

[14] Deslippe A.L., Soanes A., Bouchaud C.C., Beckenstein H., Slim M., Plourde H., Cohen T.R. (2023). Barriers and facilitators to diet, physical activity and lifestyle behavior intervention adherence: A qualitative systematic review of the literature. Int. J. Behav. Nutr. Phys. Act..

[15] Swindle T., Curran G.M., Johnson S.L. (2019). Implementation Science and Nutrition Education and Behavior: Opportunities for Integration. J. Nutr. Educ. Behav..

